# Effects of hemodialysis on plasma oxylipins

**DOI:** 10.14814/phy2.14447

**Published:** 2020-06-19

**Authors:** Benjamin Gollasch, Guanlin Wu, Inci Dogan, Michael Rothe, Maik Gollasch, Friedrich C. Luft

**Affiliations:** ^1^ Experimental and Clinical Research Center (ECRC), a joint institution between the Charité University Medicine and Max Delbrück Center (MDC) for Molecular Medicine Berlin‐Buch Germany; ^2^ HELIOS Klinikum Berlin‐Buch Berlin Germany; ^3^ Max Delbrück Center for Molecular Medicine (MDC) in the Helmholtz Association Berlin Germany; ^4^ LIPIDOMIX GmbH Berlin Germany; ^5^ Department of Geriatrics University of Greifswald University District Hospital Wolgast Greifswald Germany

**Keywords:** dialysis, eicosanoids, fatty acids, lipidomics, oxylipins

## Abstract

Chronic kidney disease (CKD) is an important risk factor for cardiovascular and all‐cause mortality. Survival rates among end‐stage renal disease (ESRD) hemodialysis patients are poor and most deaths are related to cardiovascular disease. Oxylipins constitute a family of oxygenated natural products, formed from fatty acid by pathways involving at least one step of dioxygen‐dependent oxidation. They are derived from polyunsaturated fatty acids (PUFAs) by cyclooxygenase (COX) enzymes, by lipoxygenases (LOX) enzymes, or by cytochrome P450 epoxygenase. Oxylipins have physiological significance and some could be of regulatory importance. The effects of decreased renal function and dialysis treatment on oxylipin metabolism are unknown. We studied 15 healthy persons and 15 CKD patients undergoing regular hemodialysis treatments and measured oxylipins (HPLC‐MS lipidomics) derived from cytochrome P450 (CYP) monooxygenase and lipoxygenase (LOX)/CYP ω/(ω‐1)‐hydroxylase pathways in circulating blood. We found that all four subclasses of CYP epoxy metabolites were increased after the dialysis treatment. Rather than resulting from altered soluble epoxide hydrolase (sEH) activity, the oxylipins were released and accumulated in the circulation. Furthermore, hemodialysis did not change the majority of LOX/CYP ω/(ω‐1)‐hydroxylase metabolites. Our data support the idea that oxylipin profiles discriminate ESRD patients from normal controls and are influenced by renal replacement therapies.

## Graphical abstract

To our knowledge, this is the first study to assess the impact of single hemodialysis treatment oxylipins in plasma using large‐scale lipidomics. We confirmed our hypothesis that the oxylipins status is influenced by hemodialysis treatment. Our data demonstrate that all four subclasses of CYP epoxy‐metabolites and a number of LOX/CYP ω/(ω‐1)‐hydroxylase metabolites are increased by the treatment. Moreover, ESRD patients undergoing regular dialysis show marked differences in plasma oxylipin profiles, that is, specific signatures, compared to normal control subjects.

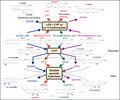

## 1 INTRODUCTION

**Figure 1:**
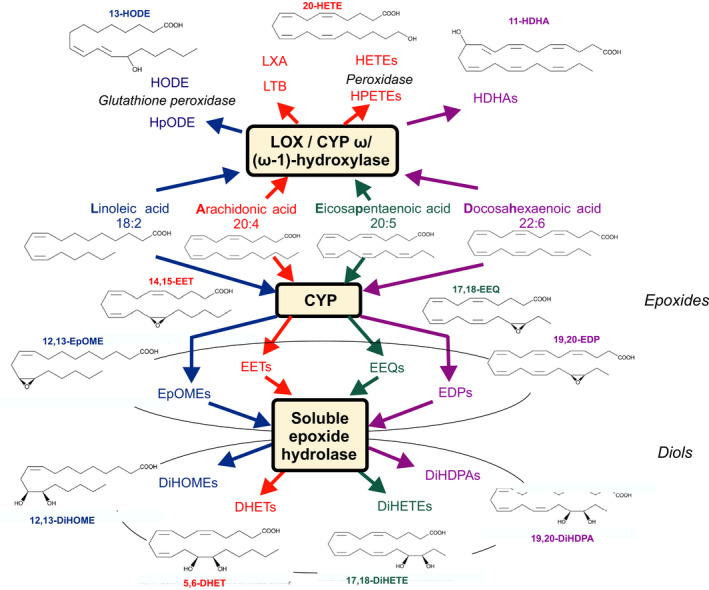
Cytochrome P450 epoxygenase (CYP) and 12‐ and 15‐lipoxygenase (LOX)/ CYP (omega‐1)‐hydroxylase pathways evaluated in response hemodialysis. Linoleic (LA), arachidonic (AA), eicosapentaenoic (EPA), and docosahexaenoic acids (DHA) are converted to epoxyoctadecenoic acids (EpOMEs, e.g., 12,13‐EpOME), epoxyeicosatrienoic acid (EETs), epoxyeicosatetraenoic acids (EEQs), and epoxydocosapentaenoic acids (EDPs) by CYP epoxygenase, respectively. EpOMEs, EETs, EEQs, and EDPs primary metabolic fate is conversion to dihydroxyctadecenoic acids (DiHOMEs, e.g., 12, 13‐DiHOME), dihydroxyeicosatrienoic acids (DHETs, e.g., 5,6‐DHET), dihydroxyeicosatetraenoic acids (DiHETEs, e.g., 5,6‐DiHETE, 17,18‐DiHETE) and dihydroxydocosapentaenoic acids (DiHDPAs, 19, 20‐DiHDPA), respectively, by the soluble epoxide hydrolase (sEH) enzyme. LA, AA, EPA, and DHA are converted to hydroperoxylinoleic acids (HpODEs), hydroxyoctadecadienoic acids (HODEs), leukotriene B (LTB), lipoxin A (LXA), hydroxydocosahexaenoic acids (HDHAs), hydroperoxyeicosatetraenoic acids (HPETEs), and hydroxyeicosatetraenoic acids (HETEs) by LOX, CYP omega/(omega‐1)‐hydroxylase and peroxidase pathways. The metabolites measured within these pathways track the changes observed in LA, AA, EPA, and DHA, respectively

Chronic kidney disease (CKD) is an important risk factor for cardiovascular and all‐cause mortality. The 5‐year survival rate among end‐stage renal disease (ESRD) hemodialysis patients is nearly 50% (McGill, 2019) and most of these deaths are related to cardiovascular disease (CVD), making ESRD a catastrophic risk factor (Luft, 2000). Conceivably, the hemodialysis treatment per se has a counterproductive effect on cardiovascular risk. Oxylipins are a superclass of lipid mediators with potent biological activities. They are derived from the oxidation of polyunsaturated fatty acids (PUFA). In addition to the well‐known eicosanoids derived from arachidonic acid (C20:4 n–6, AA), recent developments in lipidomics methodologies have raised awareness of, and interest in, the many hitherto unknown oxylipins. These products include octadecanoids derived from linoleic acid (C18:2 n–6, LA) and α‐linolenic acid (C18:3 n–3, ALA), eicosanoids derived from dihomo‐γ‐linolenic acid (C20:3 n–6), eicosapentanoic acid (C20:5 n–3, EPA), and docosanoids derived from adrenic acid (C22:4 n–6, AdA) or docosahexaenoic acid (22:6 n–3, DHA) (Gabbs, 2015). Oxylipins are primarily produced via the cytochromes P450 (CYP) monooxygenase, cyclooxygenase (COX), and lipoxygenase (LOX)/CYP ω/(ω‐1)‐hydroxylase pathways (Figure 1), resulting in the formation of mono‐, di‐, and trihydroxy fatty acids, epoxy fatty acids, prostaglandins, thromboxanes, lipoxins, resolvins, and maresin (Gabbs, 2015). Epoxyoctadecenoic acids (EpOMEs), epoxyeicosatrienoic acids (EETs), epoxyeicosatetraenoic acids (EEQs), and epoxydocosapentaenoic acids (EDPs) represent the principal subclasses of CYP epoxygenase products. The primary metabolic fate of EETs (Figure 1), EpOMEs, EEQs, and EDPs in many cells is conversion into DHETs, DiHOMEs, DiHETEs, and DiHDPAs by the soluble epoxide hydrolase enzyme (sEH) (Spector, 2004).

Oxylipins are not only important in normal physiology, but can also have detrimental effects (for review see (Gabbs, 2015; Nayeem, 2018; Tourdot, Ahmed, & Holinstat, 2014)). They could exhibit either beneficial or harmful cardiovascular effects (Afshinnia, 2018; Fan & Roman, 2017; Schunck, 2018). Oxylipin profiling of human plasma in uremic ESRD patients before fistula construction surgery has been reported (Hu, 2018). The authors identified decreases in 5,6‐dihydroxyeicosatrienoic acid (5,6‐DHET) and 5‐hydroxyeicosatetraenoic acid (5‐HETE), but increases in 9,10‐EpOME and 12,13‐EpOME as key markers that discriminated between ESRD patients and controls. The products are biologically active. For example, EETs and DHETs are considered candidates for vasodilatory endothelium‐derived hyperpolarizing factors (EDHFs) (Campbell, 1996), whose release is triggered by Ca^2+^ and shear stress via the CYP pathway (Campbell & Fleming, 2010; Graber, Alfonso, & Gill, 1997). While EpOMEs and their diols decrease cardiac postischemic functional recovery (Bannehr, 2019a), 5‐HETE stimulates neutrophil chemotaxis and degranulation, (Goetzl, 1980; Stenson & Parker, 1980; Valone, 1980) and inhibits endothelial PGI2 production with consecutive effects on platelet aggregation and vasomotor tone (Gordon, Gordon, & Spector, 1991). Hemodialysis treatment per se increases levels of plasma 6‐keto PGF1 alpha and PGF2 isoprostanes, which may also play a role in platelet aggregation and vasodilation (Kim, 2004; Kovac, 1992). Whether or not the hemodialysis treatment itself affects oxylipin profiles is unknown. We tested the hypothesis that oxylipin profiles differ in normal subjects and ESRD patients and that the hemodialysis treatment per se would affect plasma concentrations.

## 2 METHODS

The Charité University Medicine Institutional Review Board approved this registered study (ClinicalTrials.gov, Identifier: NCT03857984). In total, 15 healthy volunteers (6 male and 9 female) and 15 ESRD patients (7 men and 8 women) participated in the study (Table 1). Inclusion criteria for the group of CKD patients were: history of renal failure requiring hemodialysis/hemofiltration therapy, age over 18 years, the ability to consent, and written consent of the study participant. The patients in the group CKD were diagnosed for the following conditions: diabetes mellitus (4 patients), hypertension (3 patients), membranous glomerulonephritis (2 patients), ADPKD (autosomal dominant polycystic kidney disease) (1 patient), other or unknown (5 patients). Exclusion criteria for healthy volunteers were: age under 18 years, chronic illness requiring any medication, pregnancy, inability to follow simple instructions, relevant or severe abnormalities in medical history, or physical examination (Gollasch, 2020). Patients underwent thrice weekly dialysis, which lasted from 3 hr 45 min to 5 hr, based on high flux AK 200 dialyzers (Gambro GmbH, Hechingen, Germany).

**Table 1:** Characteristics of hemodialysis (HD) patients and control subjects (*n* = 15 each)

	HD patients	Controls
Age (years)	50 ± 18	47 ± 12
Sex		
Male (*n*)	7	6
Female (*n*)	8	9
Body mass index (kg/m^2^)	24.8 ± 3.4	24.7 ± 4.6
Race (*n*)		
	Caucasian = 14	Caucasian = 14
	Black = 1	Asian = 1
		
Cause of end‐stage renal disease		
Diabetes (*n*)	4	
Hypertension (*n*)	3	
Membranous glomerulonephritis (*n*)	2	
ADPKD (*n*)	1	
Other or unknown	5	
Complications		
Cardiovascular (*n*)	2	
Cerebrovascular (*n*)	1	
Peripheral artery disease (*n*)	3	

Note

Data are presented as mean ± *SD* or frequencies.

Venous blood was collected in each healthy subject by subcutaneous arm vein puncture in the sitting position. In the group of dialyzed patients (CKD group), all the blood samples were collected on the fistula arm right before beginning of the dialysis (pre‐HD) and at the end of the dialysis (5–15 min before termination, post‐HD). All blood samples were obtained by 4°C precooled EDTA vacuum extraction tube systems. Cells were separated from plasma by centrifugation for 10 min at 1,000–2,000 g using a refrigerated centrifuge. Following centrifugation, supernatant plasma was immediately transferred into clean polypropylene tubes using an Eppendorf pipette. The samples were maintained at 2–8°C while handling. Aliquots (0.5 ml) were then stored at –80°C until further processing and extraction. Overall, the processing took no longer than 10 min. All samples were analyzed for free and total plasma oxylipins. Oxylipins were determined by high‐performance liquid chromatography mass spectrometry (HPLC‐MS) spectrometry described in (Fischer, 2014) (Gollasch, 2019a). Descriptive statistics were calculated and variables were examined for meeting assumptions of normal distribution without skewness and kurtosis. We used the Shapiro–Wilk test to determine if they were normally distributed. In order to determine statistical significance, a two‐tailed *t* test or Mann–Whitney test were used to compare values of CKD versus control groups. Homogeneity of variances was asserted using Levene's test. Paired *t* test or paired Wilcoxon test were used to compare pre‐HD versus post‐HD values. In order to determine the statistical significance between the four classes of epoxy metabolites hydrolyzed to appear in the circulation, Friedman's test was used followed by applying Dunn's multiple comparison test (Gollasch, 2019a, 2019b). The .05 level of significance (*p*) was chosen. All data are presented as mean ± *SD*. All statistical analyses were performed using SPSS Statistics software (IBM Corporation, Armonk, NY, USA).

## 3 RESULTS

### 3.1 Clinical characteristics

Table 1 shows the demographics of the ESRD hemodialysis (HD) patients and control subjects. The results show that age and body mass indices between HD patients and the healthy subjects were not different (*p* > .05 each). Subjects were Caucasians, with the exception of one African and one Asian subject in each group. The ESRD patients had diabetes mellitus, hypertension, membranous glomerulonephritis, ADPKD, and other or unknown disease, as underlying causes. Major cardiovascular complications were cardiovascular and cerebrovascular events, and peripheral artery disease.

### 3.2 Oxylipins in ESRD

We first determined the total levels of oxylipins in plasma of the HD patients (Table 2) and compared the results with the healthy control subjects. We were particularly interested in 9,10‐EpOME, 12,13‐EpOME, 5,6‐DHET, and 5‐HETE, which have been recently identified as the key markers to discriminate uremic ESRD patients from controls (Hu, 2018). Our results confirm these findings. However, we also detected increased 5,6‐EET, 8,9‐EET, 11,12‐EET, 14,15‐EET, 5,6‐EEQ, 11,12‐EEQ, 14,15‐EEQ, 17,18‐EEQ, 14,15‐DiHETE, 7,8‐EDP, 10,11‐EDP, 16,17‐EDP, 19,20‐EDP, 5‐HEPE, 12‐HEPE, and 19‐HEPE levels in our ESRD patients undergoing regular hemodialysis treatments, compared to the control subjects (Table 2A). Moreover, 13‐HODE, 9,10‐DiHOME, 8,9‐DHET, 11,12‐DHET, 14,15‐DHET, 8‐HETE, 9‐HETE, 11‐HETE, 12‐HETE, 15‐HETE, 16‐HETE, 19‐HETE, 20‐HETE, 8‐HEPE, 9‐HEPE, 15‐HEPE, 18‐HEPE, 4‐HDHA, 7‐HDHA, 8‐HDHA, 10‐HDHA, 11‐HDHA, 13‐HDHA, 14‐HDHA, 16‐HDHA, 17‐HDHA,and 20‐HDHA levels were decreased in our ESRD patients, compared to controls. Our dialysis patients showed 12,13‐DiHOME, 8,9‐EEQ, 5,6‐DiHETE, 8,9‐DiHETE, 11,12‐DiHETE, 17,18‐DiHETE, 13,14‐EDP, 7,8‐DiHDPA, 10,11‐ DiHDPA, 13,14‐ DiHDPA, 16,17‐ DiHDPA, 19,20‐ DiHDPA, 17‐HETE, 18‐HETE, 12‐HpETE, 20‐HEPE, 21‐HDHA, and 22‐HDHA levels, which were similar to values observed in the control group. Metabolites in free state may have been contributed to changes of total 9,10‐DiHOME, 11‐HETE, 12‐HETE, or 12‐HEPE levels (Table 2B) since both total and free oxylipins showed similar data grouping effects (right columns in Table 2A and B). With exception of decreased PGE2 and PGD2 levels (Table 2B), our CKD patients showed similar or nondetectable levels of TXB2, 11‐dehydro TXB2, 15‐keto‐PGE2, 13,14‐dihydro‐15‐keto‐PGE2, 13,14‐dihydro‐15‐keto‐PGD2, PGJ2, 15‐deoxy‐delta 12,14‐PGJ2, PGF2a, 8‐iso‐PGF2a, TXB3, 11‐dehydro TXB3, PGE3, 6(S)‐LXA4, LXA4, 15(R)‐LXA4, LXB4, LXA5, MAR 1, 7‐epi‐MAR1, RvD1, 17(R)‐RvD1, RvD2, RvD3, RvD5, and RvE1, compared to healthy controls (detection level of 0.05 ng/g, each).

**Table 2:** Comparison of oxylipins between control subjects versus. CKD patients before hemodialysis (HD) (*n* = 15)

Amount ng/mL	Control (Mean ± *SD*)	HD (mean ± *SD*)	*p*‐value *t*‐Test (^#^Mann‐Whitney Test)	Data grouping effect
A. Total oxylipins in plasma.			
9,10‐EpOME	17.22 ± 11.11	57.77 ± 53.82	.001^#^	HD > Control
12,13‐EpOME	19.42 ± 16.22	57.80 ± 58.55	.004^#^	
5,6‐EET	15.66 ± 7.34	47.09 ± 53.73	.013^#^	
8,9‐EET	4.97 ± 2.07	13.12 ± 13.52	.009^#^	
11,12‐EET	2.02 ± 064	7.29 ± 8.74	<.001^#^	
14,15‐EET	8.02 ± 3.62	35.11 ± 39.14	.001^#^	
5,6‐EEQ	7.87 ± 5.32	28.3 ± 32.4	.028	
11,12‐EEQ	0.42 ± 0.27	1.48 ± 1.60	.019^#^	
14,15‐EEQ	0.32 ± 0.20	1.22 ± 1.37	.011^#^	
17,18‐EEQ	1.00 ± 0.68	4.45 ± 4.74	.002^#^	
14,15‐DiHETE	0.02 ± 0.01	0.04 ± 0.09	.041^#^	
7,8‐EDP	1.53 ± 0.52	3.65 ± 2.36	.002^#^	
10,11‐EDP	0.33 ± 0.19	0.75 ± 0.54	.005^#^	
16,17‐EDP	1.33 ± 0.48	5.34 ± 4.55	<.001^#^	
19,20‐EDP	1.56 ± 0.55	7.75 ± 5.99	<.001^#^	
5‐HEPE	1.07 ± 0.62	1.12 ± 2.19	.023^#^	
12‐HEPE	1.09 ± 0.65	1.33 ± 3.32	.002^#^	
19‐HEPE	0.77 ± 0.31	1.80 ± 5.06	.021^#^	
13‐HODE	39.08 ± 13.32	30.44 ± 14.64	.041^#^	HD < Control
9,10‐DiHOME	5.05 ± 3.17	4.07 ± 4.37	.041^#^	
5,6‐DHET	1.60 ± 0.76	0.85 ± 0.45	.004^#^	
8,9‐DHET	1.42 ± 051	1.10 ± 1.25	.016^#^	
11,12‐DHET	0.41 ± 0.15	0.31 ± 0.16	.019^#^	
14,15‐DHET	0.55 ± 0.24	0.32 ± 0.10	.003	
5‐HETE	7.22 ± 3.21	4.25 ± 1.40	.001^#^	
8‐HETE	5.60 ± 2.41	3.16 ± 4.49	.003	
9‐HETE	6.60 ± 2.23	3.78 ± 1.38	<.001	
11‐HETE	9.95 ± 2.73	5.05 ± 1.53	<.001	
12‐HETE	11.82 ± 4.23	6.52 ± 3.42	<.001^#^	
15‐HETE	17.22 ± 5.36	8.82 ± 3.30	<.001	
16‐HETE	1.39 ± 0.61	0.75 ± 0.25	<.001^#^	
19‐HETE	1.10 ± 0.51	0.58 ± 0.22	<.001^#^	
20‐HETE	0.98 ± 0.30	0.72 ± 0.26	.019	
8‐HEPE	0.50 ± 0.28	0.46 ± 1.04	.001^#^	
9‐HEPE	0.53 ± 0.29	0.48 ± 1.05	.001^#^	
15‐HEPE	0.78 ± 0.44	0.66 ± 1.40	<.001^#^	
4‐HDHA	2.39 ± 0.95	1.61 ± 1.32	.009^#^	
7‐HDHA	1.04 ± 0.47	0.79 ± 0.88	.045^#^	
8‐HDHA	1.27 ± 0.55	1.02 ± 1.29	.026^#^	
10‐HDHA	1.75 ± 0.61	1.00 ± 1.00	.001^#^	
11‐HDHA	1.33 ± 0.57	0.97 ± 1.14	.013^#^	
13‐HDHA	1.44 ± 0.54	0.90 ± 0.80	.002^#^	
14‐HDHA	2.27 ± 0.81	1.18 ± 1.33	.001^#^	
16‐HDHA	1.14 ± 0.40	0.76 ± 0.84	.002^#^	
17‐HDHA	3.02 ± 0.98	1.85 ± 1.83	.001^#^	
20‐HDHA	4.73 ± 1.78	2.90 ± 2.48	.002^#^	
12,13‐DiHOME	3.76 ± 1.67	3.18 ± 2.56	.267^#^	No effect or not significant
8,9‐EEQ	0.66 ± 0.40	1.85 ± 2.03	.098^#^	
5,6‐DiHETE	0.25 ± 0.15	0.38 ± 0.69	.461^#^	
8,9‐DiHETE	0.02 ± 0.01	0.05 ± 0.14	.102^#^	
11,12‐DiHETE	0.01 ± 0.01	0.02 ± 0.07	.074^#^	
17,18‐DiHETE	0.10 ± 0.04	0.22 ± 0.48	.838^#^	
13,14‐EDP	0.20 ± 0.11	0.45 ± 0.40	.148^#^	
7,8‐DiHDPA	0.24 ± 0.13	0.28 ± 0.37	.412^#^	
10,11‐DiHDPA	0.05 ± 0.02	0.05 ± 0.09	.260^#^	
13,14‐DiHDPA	0.04 ± 0.01	0.04 ± 0.05	.130^#^	
16,17‐DiHDPA	0.10 ± 0.04	0.11 ± 0.11	.285^#^	
19,20‐DiHDPA	0.57 ± 0.25	0.90 ± 0.23	.935^#^	
17‐HETE	0.25 ± 0.07	0.30 ± 0.23	.967^#^	
18‐HETE	0.72 ± 0.36	0.56 ± 0.24	.187^#^	
12‐HpETE	6.23 ± 3.23	12.32 ± 10.44	.067^#^	
20‐HEPE	0.13 ± 0.07	0.35 ± 0.81	.775^#^	
21‐HDHA	0.70 ± 0.23	1.53 ± 3.35	.744^#^	
22‐HDHA	0.21 ± 0.07	0.63 ± 1.55	.967^#^	
B. Free oxylipins in plasma.				
13,14‐EDP	0.01 ± 0.01	0.02 ± 0.02	.019^#^	HD > Control
9‐HEPE	0.04 ± 0.03	0.08 ± 0.25	.017^#^	
12‐HEPE	1.65 ± 1.25	3.26 ± 9.64	.015^#^	
18‐HEPE	0.38 ± 0.20	0.58 ± 1.67	.001^#^	
10‐HDHA	0.20 ± 0.10	0.23 ± 0.50	.029^#^	
16‐HDHA	0.06 ± 0.05	0.12 ± 0.37	.007^#^	
17‐HDHA	0.59 ± 0.28	0.85 ± 2.33	,004^#^	
9,10‐DiHOME	1.65 ± 2.14	0.44 ± 0.41	.046	HD < Control
5,6‐EET	0.37 ± 0.13	0.23 ± 0.10	.003	
11,12‐EET	0.06 ± 0.04	0.03 ± 0.02	.013^#^	
14,15‐DiHETE	0.03 ± 0.03	0.02 ± 0.05	.008^#^	
17,18‐DiHETE	0.27 ± 0.34	0.12 ± 0.20	.003^#^	
9‐HETE	0.12 ± 0.04	0.09 ± 0.12	.013^#^	
11‐HETE	0.74 ± 0.22	0.24 ± 0.12	<.001	
12‐HETE	9.61 ± 6.22	4.44 ± 5.60	.006^#^	
15‐HETE	1.19 ± 0.85	0.41 ± 0.22	<.001^#^	
15‐HEPE	0.14 ± 0.09	0.13 ± 0.35	.001^#^	
13‐HDHA	0.11 ± 0.07	0.10 ± 0.20	.022^#^	
14‐HDHA	1.80 ± 0.96	1.30 ± 2.23	.017^#^	
TXB2	0.38 ± 0.19	0.14 ± 0.16	.003^#^	
PGE2	0.09 ± 0.04	0.04 ± 0.03	.001^#^	
PGD2	0.05 ± 0.02	0.03 ± 0.02	.012^#^	
13‐HODE	7.68 ± 6.28	5.05 ± 5.27	.098^#^	No effect or not significant
9,10‐EpOME	0.70 ± 0.59	1.73 ± 2.01	.098^#^	
12,13‐EpOME	0.78 ± 0.70	1.59 ± 1.89	.254^#^	
12,13‐DiHOME	9.02 ± 14.34	2.93 ± 2.75	.080^#^	
8,9‐EET	0.02 ± 0.02	0.03 ± 0.03	.180	
14,15‐EET	0.31 ± 0.19	0.39 ± 0.28	.397	
5,6‐DHET	0.01 ± 0.01	0.01 ± 0.01	.856^#^	
8,9‐DHET	0.11 ± 0.12	0.09 ± 0.05	.719^#^	
11,12‐DHET	0.11 ± 0.09	0.08 ± 0.04	.555^#^	
14,15‐DHET	0.05 ± 0.02	0.04 ± 0.01	.067	
5,6‐EEQ	0.37 ± 0.12	0.32 ± 0.19	.688	
8,9‐EEQ	0.09 ± 0.08	0.35 ± 0.73	.241^#^	
11,12‐EEQ	0.02 ± 0.01	0.05 ± 0.12	1.000^#^	
14,15‐EEQ	0.09 ± 0.08	0.22 ± 0.59	.222^#^	
17,18‐EEQ	0.27 ± 0.22	0.75 ± 2.15	.467^#^	
5,6‐DiHETE	0.01 ± 0.01	0.01 ± 0.02	.098^#^	
8,9‐DiHETE	0.01 ± 0.01	0.01 ± 0.03	.896^#^	
11,12‐DiHETE	0.01 ± 0.01	0.01 ± 0.01	.047^#^	
7,8‐EDP	0.15 ± 0.8	0.15 ± 0.21	.235^#^	
10,11‐EDP	0.01 ± 0.01	0.03 ± 0.05	.316^#^	
16,17‐EDP−233	0.04 ± 0.06	0.02 ± 0.02	.185^#^	
19,20‐EDP	0.03 ± 0.04	0.49 ± 1.43	.072^#^	
7,8‐DiHDPA	0.01 ± 0.01	0.01 ± 0.01	.650^#^	
10,11‐DiHDPA	0.01 ± 0.01	0.01 ± 0.01	.200^#^	
13,14‐DiHDPA	0.01 ± 0.01	0.01 ± 0.01	.142^#^	
16,17‐DiHDPA	0.08 ± 0.05	0.09 ± 0.07	.717^#^	
19,20‐DiHDPA	0.34 ± 0.20	0.64 ± 0.81	.363^#^	
5‐HETE	0.07 ± 0.03	0.09 ± 0.11	.892^#^	
8‐HETE	0.17 ± 0.08	0.14 ± 0.09	.294^#^	
16‐HETE	0.29 ± 0.08	0.25 ± 0.10	.282	
17‐HETE	0.05 ± 0.02	0.11 ± 0.19	.928^#^	
18‐HETE	0.01 ± 0.01	0.03 ± 0.05	.339^#^	
19‐HETE	0.04 ± 0.01	0.04 ± 0.02	.690	
20‐HETE	0.25 ± 0.10	0.35 ± 0.19	.118^#^	
12‐HpETE	0.04 ± 0.04	0.05 ± 0.04	.598	
5‐HEPE	0.05 ± 0.03	0.15 ± 0.42	.440^#^	
19‐HEPE	0.35 ± 0.42	1.45 ± 4.24	.339^#^	
20‐HEPE	0.03 ± 0.06	0.24 ± 0.50	.823^#^	
4‐HDHA	0.03 ± 0.03	0.23 ± 0.73	.821^#^	
7‐HDHA	0.01 ± 0.01	0.05 ± 0.14	.294^#^	
8‐HDHA	0.02 ± 0.02	0.07 ± 0.19	.387^#^	
11‐HDHA	0.12 ± 0.07	0.28 ± 0.74	.254^#^	
20‐HDHA	0.16 ± 0.09	0.16 ± 0.37	.004^#^	
21‐HDHA	0.39 ± 0.51	2.02 ± 5.94	.892^#^	
22‐HDHA	0.32 ± 0.43	1.10 ± 3.00	.751^#^	
15‐keto‐PGE2	*n*.d.	*n*.d.		
13,14‐dihydro−15‐keto‐PGE2	*n*.d.	*n*.d.		
13,14‐dihydro−15‐keto‐PGD2	*n*.d.	*n*.d.		
PGJ2	*n*.d.	*n*.d.		
15‐deoxy‐delta 12,14‐PGJ2	*n*.d.	*n*.d.		
PGF2a8‐iso‐PGF2a	*n*.d.	*n*.d.		
TXB3	*n*.d.	*n*.d.		
11‐dehydro TXB3	*n*.d.	*n*.d.		
PGE3	*n*.d.	*n*.d.		
6(S)‐LXA4	*n*.d.	*n*.d.		
LXA4	*n*.d.	*n*.d.		
15(R)‐LXA4	*n*.d.	*n*.d.		
LXB4	*n*.d.	*n*.d.		
LXA5	*n*.d.	*n*.d.		
MAR 1	*n*.d.	*n*.d.		
7‐epi‐MAR1	*n*.d.	*n*.d.		
RvD1	*n*.d.	*n*.d.		
17(R)‐RvD1	*n*.d.	*n*.d.		
RvD2	*n*.d.	*n*.d.		
RvD3	*n*.d.	*n*.d.		
RvD5	*n*.d.	*n*.d.		

Together, the findings indicate that ESRD is associated with an altered plasma oxylipins status namely an individual signature, which shows decreases in the majority of LOX/CYP ω/(ω‐1)‐hydroxylase‐dependent metabolites (HETEs, HEPEs, HDTAs, and 13‐HODE) and increases in all four classes of CYP epoxy metabolites (i.e., EET, EpOME, EEQ, and EDP).

### 3.3 Diol/epoxide ratios

As shown in Figure 1, the main pathway of EET, EpOME, EEQ, and EDP metabolism in many cells is conversion into DHETs, DiHOMEs, DiHETEs, and DiHDPAs by the soluble epoxide hydrolase enzyme (sEH), even though epoxy‐polyunsaturated fatty acids (EpPUFA) can also be nonenzymatically hydrolyzed into dihydroxymetabolites (DiHPUFA) (Spector, 2004). Since ESRD might have caused EET, EpOME, EEQ, and EDP production rapidly degraded to their diols, we next analyzed the sums of the individual CYP epoxy metabolites and their diols (Table 3A). We found that ESRD was associated with increased levels of the majority of those CYP metabolites (Table 3A). To provide insights into possible mechanisms underlying this increase, we calculated diol/epoxide ratios of the epoxy metabolites (Table 3B and C **)**. We found that the four classes of epoxy metabolites are unequally hydrolyzed to appear in the circulation (Friedman's test, *p* < .05). We found that EpOMEs and EDPs are better metabolized into their diols (ratio of DiHOMEs/EPOMEs and DiHDPAs/EDPs; 0.156 ± 0.301 and 0.114 ± 0.141, respectively; Dunn's multiple comparison test, *p* > .05) than EETs and EEQs (ratios of those diols/epoxy metabolites, 0.047 ± 0.046 and 0.027 ± 0.031, respectively; Dunn's multiple comparison test, *p* > .05) (Table 3B). In fact, the following order of ratios was identified: DiHOMEs/EpOMEs = DiHDPA/EDPs > DHETs/EETs = DiHETEs/EEQs (Dunn's multiple comparison test, *p* < .05). This pattern was also found for the individual metabolites in vivo, as shown (Table 3C **)**. Together, the findings indicate that CYP epoxy metabolites are released and accumulated in the circulation of ESRD HD patients, compared to controls, with epoxy metabolite substrate classes unequally hydrolyzed by sEH in vivo.

**Table 3:** Comparison of oxylipins and their ratios between control subjects versus. CKD patients before hemodialysis (HD) (*n* = 15)

Epoxides or Diols (ng/mL)	Control (Mean ± *SD*)	HD (Mean ± *SD*)	*p*‐value Mann‐Whitney test	Data grouping effect
A. Concentrations of individual total epoxides plus their respective diols in plasma.				
9,10‐ EpOME + 9,10‐DiHOME	22.27 ± 12.62	61.84 ± 53.16	.0025	HD > Control
12,13‐EpOME + 12,13‐DiHOME	23.18 ± 17.10	60.97 ± 58.41	.0062	
5,6‐EET + 5,6‐DHET	17.25 ± 7.548	47.94 ± 53.82	.0213	
8,9‐EET + 8,9‐DHET	6.213 ± 2.207	14.22 ± 13.81	.0421	
11,12 EET + 11,12‐DHET	2.427 ± 0.7535	7.601 ± 8.773	.0006	
14,15‐EET + 14,15‐DHET	8.574 ± 3.654	35.43 ± 39.17	.0014	
5,6‐EEQ + 5,6‐DiHETE	8.119 ± 5.428	28.67 ± 32.50	.0344	
11,12‐EEQ + 11,12‐DiHETE	0.4307 ± 0.2757	1.502 ± 1.610	.0263	
14,15‐EEQ + 14,15‐DiHETE	0.3400 ± 0.2008	1.260 ± 1.377	.0170	
17,18‐EEQ + 17,18‐DiHETE	1.101 ± 0.7089	4.671 ± 4.833	.0042	
7,8‐EDP + 7,8‐DiHDPA	1.766 ± 0.5502	3.930 ± 2.449	.0045	
10,11‐EDP + 10,11‐DiHDPA	0.3813 ± 0.1933	0.8080 ± 0.5495	.0152	
16,17‐EDP + 16,17‐DiHDPA	1.425 ± 0.4758	5.445 ± 4.546	.0003	
19,20‐EDP + 19,20‐DiHDPA	2.126 ± 0.6048	8.650 ± 6.002	<.0001	
8,9‐EEQ + 8,9‐DiHETE	0.6840 ± 0.4120	1.901 ± 2.067	.1103	No effect or not significant
13,14‐EDP + 13,14‐DiHDPA	0.2453 ± 0.1193	0.4880 ± 0.4005	.1581	
**B**: Ratios estimated using total concentrations of epoxides and diols in plasma.				
Ratio (9,10‐DiHOME + 12,13‐DiHOME) / (9,10‐EpOME + 12,13‐EpOME)	0.3009 ± 0.1402	0.1559 ± 0.3014	.0008	HD < Control
Ratio (5,6‐DHET + 8,9‐DHET + 11,12‐DHET + 14,15‐DHET) / (5,6‐EET + 8,9‐EET + 11,12 EET + 14,15‐EET)	0.1406 ± 0.07617	0.04724 ± 0.04648	<.0001	
Ratio (5,6‐DiHETE + 8,9‐DiHETE + 11,12‐DiHETE + 14,15‐DiHETE + 17,18‐DiHETE) / (5,6‐EEQ + 8,9‐EEQ + 11,12‐EEQ + 14,15‐EEQ + 17,18‐EEQ)	0.04801 ± 0.02220	0.02745 ± 0.03105	.0028	
Ratio (7,8‐DiHDPA + 10,11‐DiHDPA + 13,14‐DiHDPA + 16,17‐DiHDPA + 19,20‐DiHDPA) / (7,8‐EDP + 10,11‐EDP + 13,14‐EDP + 16,17‐EDP + 19,20‐EDP)	0.2242 ± 0.1244	0.1145 ± 0.1411	.0032	

Ratios	Control (Mean ± *SD*)	HD (Mean ± *SD*)	*p*‐value Mann‐Whitney test	Data grouping effect
C. Ratios estimated using individual total concentrations of epoxides and their diols in plasma.				
9,10‐DiHOME / 9,10‐EpOME	0.3487 ± 0.1679	0.1792 ± 0.3752	.0003	HD < Control
12,13‐DiHOME / 12,13‐EpOME	0.2567 ± 0.1225	0.1320 ± 0.2280	.0012	
5,6‐DHET / 5,6‐EET	0.1156 ± 0.06784	0.03753 ± 0.03869	.0002	
8,9‐DHET / 8,9‐EET	0.2790 ± 0.1382	0.1206 ± 0.1024	.0006	
11,12‐DHET / 11,12‐EET	0.2092 ± 0.05280	0.07844 ± 0.06571	.0002	
14,15‐DHET / 14,15‐EET	0.08377 ± 0.05311	0.02045 ± 0.02099	<.0001	
5,6‐DiHETE / 5,6‐EEQ	0.03749 ± 0.01789	0.01923 ± 0.02031	.0028	
8,9‐DiHETE / 8,9‐EEQ	0.04396 ± 0.02798	0.02663 ± 0.04312	.0021	
11,12‐DiHETE / 11,12‐EEQ	0.03184 ± 0.01914	0.01553 ± 0.02861	.0008	
14,15‐DiHETE / 14,15‐EEQ	0.08126 ± 0.04245	0.04268 ± 0.05904	.0014	
17,18‐DiHETE / 17,18‐EEQ	0.1335 ± 0.06799	0.07081 ± 0.08064	.0070	
7,8‐DiHDPA / 7,8‐EDP	0.1699 ± 0.1156	0.09037 ± 0.09216	.0070	
10,11‐DiHDPA / 10,11‐EDP	0.2078 ± 0.1351	0.08913 ± 0.1152	.0008	
13,14‐DiHDPA / 13,14‐EDP	0.2631 ± 0.1276	0.1662 ± 0.1892	.0202	
16,17‐DiHDPA / 16,17‐EDP	0.08331 ± 0.04474	0.03211 ± 0.03420	.0004	
19,20‐DiHDPA / 19,20‐EDP	0.4071 ± 0.2295	0.1934 ± 0.2658	.0025	

### 3.4 Effects of hemodialysis

The effects of hemodialysis treatment on plasma oxylipins in ESRD HD patients are summarized (Table 4). The data demonstrate an increase in the majority of epoxy metabolites, including 9,10‐EpOME, 12,13‐EpOME, 9,10‐DiHOME, 12,13‐DiHOME, 5,6‐EET, 14,15‐EET, 8,9‐DHET, 11,12‐DHET, 8,9‐EEQ, 11,12‐EEQ, 14,15‐EEQ, 17,18‐EEQ, 14,15‐DiHETE, 17,18‐DiHETE, 7,8‐EDP, 10,11‐EDP, 13,14‐EDP, 16,17‐EDP, 19,20‐EDP, 7,8‐DiHDPA, 10,11‐DiHDPA, 13,14‐DiHDPA, 16,17‐DiHDPA, and 19,20‐DiHDPA (Table 4A). Moreover, hemodialysis also increased several LOX/CYP ω/(ω‐1)‐hydroxylase metabolites, such as 13‐HODE, 20‐HETE, 5‐HEPE, 15‐HEPE, 18‐HEPE, 19‐HEPE, 7‐HDHA, and 11‐HDHA. No changes occurred in the levels of other CYP and LOX/CYP ω/(ω‐1)‐hydroxylase metabolites (Table 4A). No level variations of free oxylipins were found in response to hemodialysis, with the exception of 9,10‐DiHOME, 11,12‐EET, 19,20‐DiHDPA, and 12‐HpETE (Table 4B). While TXB2, PGE2, and PGD2 levels did not change by hemodialysis, this treatment did not cause accumulation of detectable levels of 11‐dehydro TXB2, 15‐keto‐PGE2, 13,14‐dihydro‐15‐keto‐PGE2, 13,14‐dihydro‐15‐keto‐PGD2, PGJ2, 15‐deoxy‐delta 12,14‐PGJ2, PGF2a, 8‐iso‐PGF2a, TXB3, 11‐dehydro TXB3, PGE3, 6(S)‐LXA4, LXA4, 15(R)‐LXA4, LXB4, LXA5, MAR 1, 7‐epi‐MAR1, RvD1, 17(*R*)‐RvD1, RvD2, RvD3, RvD5, and RvE1 in plasma (Table 4B **)** at a detection level of 0.05 ng/g, each.

**Table 4:** Effects of hemodialysis on oxylipins in the CKD patients before (pre‐HD) and at cessation (post‐HD) of hemodialysis (*n* = 15 each)

Amount ng/ml	Pre‐HD (Mean ± *SD*)	Post‐HD (mean ± *SD*)	*p*‐value Paired *t*‐Test (^#^paired Wilcoxon Test)	Data grouping effect
A. Total oxylipins in plasma.				
13‐HODE	30.44 ± 14.64	42.19 ± 23.88	.016^#^	Post‐HD > Pre‐HD
9,10‐EpOME	57.77 ± 53.82	145.71 ± 161.87	.041^#^	
12,13‐EpOME	57.80 ± 58.55	140.29 ± 145.06	.041^#^	
9,10‐DiHOME	4.07 ± 4.37	6.35 ± 4.81	.019^#^	
12,13‐DiHOME	3.18 ± 2.56	5.81 ± 3.71	.004^#^	
5,6‐EET	47.09 ± 53.73	127.44 ± 131.75	.035^#^	
14,15‐EET	35.11 ± 39.14	103.89 ± 115.46	.048^#^	
8,9‐DHET	1.10 ± 1.25	1.29 ± 1.49	.011^#^	
11,12‐DHET	0.31 ± 0.16	0.38 ± 0.19	.022^#^	
8,9‐EEQ	1.85 ± 2.03	5.27 ± 6.00	.019^#^	
11,12‐EEQ	1.48 ± 1.60	4.43 ± 5.59	.035^#^	
14,15‐EEQ	1.22 ± 1.37	3.56 ± 4.52	.030^#^	
17,18‐EEQ	4.45 ± 4.74	13.85 ± 18.04	.048^#^	
14,15‐DiHET	0.04 ± 0.09	0.05 ± 0.10	.019^#^	
17,18‐DiHETE	0.22 ± 0.48	0.25 ± 0.49	.006^#^	
7,8‐EDP	3.65 ± 2.36	9.67 ± 8.71	.041^#^	
10,11‐EDP	0.75 ± 0.54	1.98 ± 2.23	.041^#^	
13,14‐EDP	0.45 ± 0.40	1.33 ± 1.64	.048^#^	
16,17‐EDP	5.34 ± 4.55	17.46 ± 18.62	.041^#^	
19,20‐EDP	7.75 ± 5.99	26.39 ± 27.84	.035^#^	
7,8‐DiHDPA	0.28 ± 0.37	0.31 ± 0.40	.016^#^	
10,11‐DiHDPA	0.05 ± 0.09	0.06 ± 0.10	.011^#^	
13,14‐DiHDPA	0.04 ± 0.05	0.05 ± 0.05	.022^#^	
16,17‐DiHDPA	0.11 ± 0.11	0.13 ± 0.11	.002^#^	
19,20‐DiHDPA	0.90 ± 0.23	1.06 ± 1.17	.016^#^	
20‐HETE	0.72 ± 0.26	0.86 ± 0.26	.032	
5‐HEPE	1.12 ± 2.19	1.33 ± 2.63	.022^#^	
15‐HEPE	0.66 ± 1.40	0.78 ± 1.51	.013^#^	
18‐HEPE	1.78 ± 3.7	2.25 ± 4.58	.041^#^	
7‐HDHA	0.79 ± 0.88	0.91 ± 0.93	.048^#^	
11‐HDHA	0.97 ± 1.14	1.18 ± 1.27	.041^#^	
19‐HEPE	1.80 ± 5.06	1.62 ± 3.78	.041^#^	Pre‐HD > Post‐HD
8,9‐EET	13.12 ± 13.52	29.53 ± 27.79	.056^#^	No effect or not significant
11,12‐EET	7.29 ± 8.74	12.97 ± 11.42	.124^#^	
5,6‐DHET	0.85 ± 0.45	0.87 ± 0.40	.826^#^	
14,15‐DHET	0.32 ± 0.10	0.36 ± 0.10	.300	
5,6‐EEQ	28.3 + 32.4	87.0 + 114.0	.132	
5,6‐DiHETE	0.38 ± 0.69	0.42 ± 0.78	.272^#^	
8,9‐DiHETE	0.05 ± 0.1	0.06 ± 0.17	.221^#^	
11,12‐DiHETE	0.02 ± 0.07	0.03 ± 0.08	.109^#^	
5‐HETE	4.25 ± 1.40	4.84 ± 2.12	.213	
8‐HETE	3.16 ± 4.49	3.70 ± 1.75	.108	
9‐HETE	3.78 ± 1.38	4.83 ± 2.51	.083	
11‐HETE	5.05 ± 1.53	6.39 ± 3.21	.116	
12‐HETE	6.52 ± 3.42	7.11 ± 3.14	.158^#^	
15‐HETE	8.82 ± 3.30	10.82 ± 6.77	.109^#^	
16‐HETE	0.75 ± 0.25	0.92 ± 0.66	.221^#^	
17‐HETE	0.30 ± 0.23	0.33 ± 0.31	.363^#^	
18‐HETE	0.56 ± 0.24	0.56 ± 0.28	.638^#^	
19‐HETE	0.58 ± 0.2	0.58 ± 0.27	.925^#^	
12‐HpETE	12.32 ± 10.44	15.78 ± 19.17	.972^#^	
8‐HEPE	0.46 ± 1.04	0.54 ± 1.17	.096^#^	
9‐HEPE	0.48 ± 1.05	0.53 ± 1.08	.064^#^	
12‐HEPE	1.33 ± 3.32	1.52 ± 3.64	.064^#^	
20‐HEPE	0.35 ± 0.81	0.29 ± 0.49	.064^#^	
4‐HDHA	1.61 ± 1.32	1.94 ± 1.70	.084^#^	
8‐HDHA	1.02 ± 1.29	1.11 ± 1.32	.221^#^	
10‐HDHA	1.00 ± 1.00	1.16 ± 1.08	.056^#^	
13‐HDHA	0.90 ± 0.80	1.10 ± 0.93	.074^#^	
14‐HDHA	1.18 ± 1.33	1.42 ± 1.58	.064^#^	
16‐HDHA	0.76 ± 0.84	0.91 ± 0.92	.084^#^	
17‐HDHA	1.85 ± 1.83	2.26 ± 2.02	.084^#^	
20‐HDHA	2.90 ± 2.48	3.35 ± 3.00	.158^#^	
21‐HDHA	1.53 ± 3.35	1.29 ± 2.14	.925^#^	
22‐HDHA	0.63 ± 1.55	0.51 ± 0.81	.096^#^	
B. Free oxylipins in plasma.				
9,10‐DiHOME	0.44 ± 0.41	1.07 ± 1.12	.034^#^	Post‐HD > Pre‐HD
11,12‐EET	0.03 ± 0.02	0.04 ± 0.02	.015^#^	
19,20‐DiHDPA	0.64 ± 0.81	0.69 ± 0.74	.019^#^	
12‐HpETE	0.05 ± 0.04	0.39 ± 0.36	.027	
13‐HODE	5.05 ± 5.27	9.23 ± 7.89	.182^#^	No effect or not significant
9,10‐EpOME	1.73 ± 2.01	3.55 ± 2.62	.308^#^	
12,13‐EpOME	1.59 ± 1.89	4.28 ± 4.22	.272^#^	
12,13‐DiHOM	2.93 ± 2.75	6.10 ± 8.36	.099^#^	
5,6‐EET	0.23 ± 0.10	0.40 ± 0.32	.071^#^	
8,9‐EET	0.03 ± 0.03	0.02 ± 0.02	.347^#^	
14,15‐EET	0.39 ± 0.28	0.63 ± 0.66	.433^#^	
5,6‐DHET	0.01 ± 0.01	0.01 ± 0.01	.329	
8,9‐DHET	0.09 ± 0.05	0.10 ± 0.05	.131^#^	
11,12‐DHET	0.08 ± 0.04	0.09 ± 0.04	.306	
14,15‐DHET	0.04 ± 0.01	0.05 ± 0.02	.218	
5,6‐EEQ	0.32 ± 0.19	0.29 ± 0.34	.785	
8,9‐EEQ	0.35 ± 0.73	0.22 ± 0.26	.753^#^	
11,12‐EEQ	0.05 ± 0.12	0.02 ± 0.03	.310^#^	
14,15‐EEQ	0.22 ± 0.59	0.16 ± 0.28	.674^#^	
17,18‐EEQ	0.75 ± 2.15	0.50 ± 0.69	.071^#^	
5,6‐DiHETE	0.01 ± 0.02	0.01 ± 0.01	1.000^#^	
8,9‐DiHETE	0.01 ± 0.03	0.01 ± 0.02	.484^#^	
11,12‐DiHETE	0.01 ± 0.01	0.01 ± 0.01	.169^#^	
14,15‐DiHETE	0.02 ± 0.05	0.03 ± 0.04	.099^#^	
17,18‐DiHETE	0.12 ± 0.20	0.14 ± 0.19	.084^#^	
7,8‐EDP	0.15 ± 0.21	0.18 ± 0.17	.530^#^	
10,11‐EDP	0.03 ± 0.05	0.02 ± 0.02	.594^#^	
13,14‐EDP	0.02 ± 0.02	0.02 ± 0.01	.878^#^	
16,17‐EDP−233	0.02 ± 0.02	0.02 ± 0.02	.010^#^	
19,20‐EDP	0.49 ± 1.43	0.26 ± 0.46	.433^#^	
7,8‐DiHDPA	0.01 ± 0.01	0.01 ± 0.02	.272^#^	
10,11‐DiHDPA	0.01 ± 0.01	0.01 ± 0.01	.477^#^	
13,14‐DiHDPA	0.01 ± 0.01	0.01 ± 0.01	.218	
16,17‐DiHDPA	0.09 ± 0.07	0.10 ± 0.52	.136^#^	
5‐HETE	0.09 ± 0.11	0.08 ± 0.07	.530^#^	
8‐HETE	0.14 ± 0.09	0.14 ± 0.06	.695^#^	
9‐HETE	0.09 ± 0.12	0.09 ± 0.07	.388^#^	
11‐HETE	0.24 ± 0.12	0.25 ± 0.11	.893	
12‐HETE	4.44 ± 5.60	4.34 ± 3.23	.875^#^	
15‐HETE	0.41 ± 0.22	0.54 ± 0.29	.236	
16‐HETE	0.25 ± 0.10	0.30 ± 0.14	.209^#^	
17‐HETE	0.11 ± 0.19	0.11 ± 0.15	.530^#^	
18‐HETE	0.03 ± 0.05	0.03 ± 0.03	.875^#^	
19‐HETE	0.04 ± 0.02	0.04 ± 0.03	.068^#^	
20‐HETE	0.35 ± 0.19	0.39 ± 0.18	.695^#^	
5‐HEPE	0.15 ± 0.42	0.09 ± 0.12	.433^#^	
8‐HEPE	0.08 ± 0.23	0.07 ± 0.22	1.000^#^	
9‐HEPE	0.08 ± 0.25	0.05 ± 0.16	.754^#^	
12‐HEPE	3.26 ± 9.64	2.42 ± 7.17	1.000^#^	
15‐HEPE	0.13 ± 0.35	0.12 ± 0.28	.433^#^	
18‐HEPE	0.58 ± 1.67	0.44 ± 1.18	.814^#^	
19‐HEPE	1.45 ± 4.24	0.78 ± 1.35	.136^#^	
20‐HEPE	0.24 ± 0.50	0.09 ± 0.20	.893^#^	
4‐HDHA	0.23 ± 0.73	0.14 ± 0.40	.695^#^	
‐HDHA	0.05 ± 0.14	0.02 ± 0.05	.209^#^	
8‐HDHA	0.07 ± 0.19	0.04 ± 0.09	.272^#^	
10‐HDHA	0.23 ± 0.50	0.18 ± 0.33	.583^#^	
11‐HDHA	0.28 ± 0.74	0.18 ± 0.34	.638^#^	
13‐HDHA	0.10 ± 0.20	0.08 ± 0.12	.814^#^	
14‐HDHA	1.30 ± 2.23	1.17 ± 1.61	.638^#^	
16‐HDHA	0.12 ± 0.37	0.09 ± 0.23	.182^#^	
17‐HDHA	0.85 ± 2.33	0.63 ± 1.24	.308^#^	
20‐HDHA	0.16 ± 0.37	0.12 ± 0.16	.136^#^	
21‐HDHA	2.02 ± 5.94	1.06 ± 2.35	.754^#^	
22‐HDHA	1.10 ± 3.00	0.61 ± 1.20	.638^#^	
TXB2	0.14 ± 0.16	0.10 ± 0.08	.678^#^	
11‐dehydro TXB2	*n*.d.	*n*.d.		
PGE2	0.04 ± 0.03	0.03 ± 0.03	.540	
15‐keto‐PGE2	*n*.d.	*n*.d.		
13,14‐dihydro−15‐keto‐PGE2	*n*.d.	*n*.d.		
PGD2	0.03 ± 0.02	0.02 ± 0.01	.674^#^	
13,14‐dihydro−15‐keto‐PGD2	*n*.d.	*n*.d.		
PGJ2	*n*.d.	*n*.d.		
15‐deoxy‐delta 12,14‐PGJ2	*n*.d.	*n*.d.		
PGF2a8‐iso‐PGF2a	*n*.d.	*n*.d.		
TXB3	*n*.d.	*n*.d.		
11‐dehydro TXB3	*n*.d.	*n*.d.		
PGE3	*n*.d.	*n*.d.		
6(S)‐LXA4	*n*.d.	*n*.d.		
LXA4	*n*.d.	*n*.d.		
15(R)‐LXA4	*n*.d.	*n*.d.		
LXB4	*n*.d.	*n*.d.		
LXA5	*n*.d.	*n*.d.		
MAR 1	*n*.d.	*n*.d.		
7‐epi‐MAR1	*n*.d.	*n*.d.		
RvD1	*n*.d.	*n*.d.		
17(R)‐RvD1	*n*.d.	*n*.d.		
RvD2	*n*.d.	*n*.d.		
RvD3	*n*.d.	*n*.d.		
RvD5	*n*.d.	*n*.d.		
RvE1	*n*.d.	*n*.d.		

### 3.5 Diol/epoxide ratios

Our analysis of sums of the individual CYP epoxy metabolites and their diols (Table 5A) demonstrated increased accumulation of (9,10‐EpOME + 9,10‐DiHOME), (12,13‐EpOME + 12,13‐DiHOME), (5,6‐EET + 5,6‐DHET) (5,6‐EEQ + 5,6‐DiHETE), (8,9‐EEQ + 8,9‐DiHETE), (11,12‐EEQ + 11,12‐DiHETE), (14,15‐EEQ + 14,15‐DiHETE), (17,18‐EEQ + 17,18‐DiHETE), (7,8‐EDP + 7,8‐DiHDPA), (19,20‐EDP + 19,20‐DiHDPA), (8,9‐EET + 8,9‐DHET), (14,15‐EET + 14,15‐DHET), (10,11‐EDP + 10,11‐DiHDPA), (13,14‐EDP + 13,14‐DiHDPA) and (16,17‐EDP + 16,17‐DiHDPA). To provide insights into possible mechanisms, we calculated ratios of diols/epoxides. We found that the ratios were not influenced by hemodialysis (Table 5B and C **)**. Together, the results indicate that the CYP epoxy metabolites are rather released and accumulated in the circulation during hemodialysis treatment than resulting from altered sEH activity in vivo. Furthermore, hemodialysis treatment is insufficient to change the majority of LOX/CYP ω/(ω‐1)‐hydroxylase metabolites in ESRD patients.

**Table 5:** Effects of hemodialysis on oxylipins and their ratios in the CKD patients before (pre‐HD) and at cessation (post‐HD) of hemodialysis (*n* = 15)

Epoxides or Diols (ng/mL)	Pre‐HD (Mean ± *SD*)	Post‐HD (Mean ± *SD*)	*p*‐value Paired Wilcoxon test	Data grouping effect
A. Concentrations of individual total epoxides plus their respective diols in plasma.				
9,10‐EpOME + 9,10‐DiHOME	62.70 ± 55.05	152.05 ± 160.20	.035	Post HD > Pre‐HD
12,13‐EpOME + 12,13‐DiHOME	62.12 ± 60.44	146.11 ± 144.84	.030	
5,6‐EET + 5,6‐DHET	49.70 ± 55.40	128.30 ± 131.89	.035	
5,6‐EEQ + 5,6‐DiHETE	29.28 ± 33.64	87.44 ± 113.99	.025	
8,9‐EEQ + 8,9‐DiHETE	1.89 ± 2.14	5.33 ± 6.90	.017	
11,12‐EEQ + 11,12‐DiHETE	1.52 ± 1.67	4.46 ± 5.58	.035	
14,15‐EEQ + 14,15‐DiHETE	1.27 ± 1.43	3.60 ± 4.50	.030	
17,18‐EEQ + 17,18‐DiHETE	4.70 ± 5.01	14.10 ± 18.00	.049	
7,8‐EDP + 7,8‐DiHDPA	4.03 ± 2.51	9.97 ± 8.70	.035	
19,20‐EDP + 19,20‐DiHDPA	8.82 ± 6.19	27.45 ± 27.56	.035	
8,9‐EET + 8,9‐DHET	14.65 ± 14.23	30.82 ± 28.47	.042	
14,15‐EET + 14,15‐DHET	35.52 ± 40.42	104.25 ± 115.45	.049	
10,11‐EDP + 10,11‐DiHDPA	0.81 ± 0.57	2.05 ± 2.22	.042	
13,14‐EDP + 13,14‐DiHDPA	0.49 ± 0.41	1.37 ± 1.63	.042	
16,17‐EDP + 16,17‐DiHDPA	5.53 ± 4.70	17.58 ± 18.59	.042	
11,12‐EET + 11,12‐DHET	8.01 ± 8.96	13.36 ± 11.42	.119	No effect or not significant
**B**. Ratios estimated using total concentrations of epoxides and diols in plasma.				
Ratio (9,10‐DiHOME + 12,13‐DiHOME) / (9,10‐EpOME + 12,13‐EpOME)	0.1624 ± 0.3117	0.2179 ± 0.4314	.670	No effect or not significant
Ratio (5,6‐DHET + 8,9‐DHET + 11,12‐DHET + 14,15‐DHET) / (5,6‐EET + 8,9‐EET + 11,12 EET + 14,15‐EET)	0.0475 ± 0.0482	0.0460 ± 0.0634	.391	
Ratio (5,6‐DiHETE + 8,9‐DiHETE + 11,12‐DiHETE + 14,15‐DiHETE + 17,18‐DiHETE) / (5,6‐EEQ + 8,9‐EEQ + 11,12‐EEQ + 14,15‐EEQ + 17,18‐EEQ)	0.0279 ± 0.0322	0.0353 ± 0.0557	.626	
Ratio (7,8‐DiHDPA + 10,11‐DiHDPA + 13,14‐DiHDPA + 16,17‐DiHDPA + 19,20‐DiHDPA) / (7,8‐EDP + 10,11‐EDP + 13,14‐EDP + 16,17‐EDP + 19,20‐EDP)	0.1160 ± 0.1462	0.1369 ± 0.2039	0.502	

Ratios	Pre‐HD (Mean ± *SD*)	Post‐HD (Mean ± *SD*)	*p*‐value Paired Wilcoxon test	Data grouping effect
C. Ratios estimated using individual total concentrations of epoxides and their diols in plasma.				
9,10‐DiHOME / 9,10‐EpOME	0.1881 ± 0.3877	0.2402 ± 0.4617	.903	No effect or not significant
12,13‐DiHOME / 12,13‐EpOME	0.1361 ± 0.2361	0.1966 ± 0.4089	.583	
5,6‐DHET / 5,6‐EET	0.0379 ± 0.0401	0.0368 ± 0.0537	.296	
8,9‐DHET / 8,9‐EET	0.1207 ± 0.1062	0.1042 ± 0.1197	.268	
11,12‐DHET / 11,12‐EET	0.0712 ± 0.0616	0.0674 ± 0.0650	.670	
14,15‐DHET / 14,15‐EET	0.0206 ± 0.0218	0.0229 ± 0.0313	.463	
5,6‐DiHETE / 5,6‐EEQ	0.0196 ± 0.0210	0.0204 ± 0.0313	.426	
8,9‐DiHETE / 8,9‐EEQ	0.0272 ± 0.0447	0.0298 ± 0.0494	.670	
11,12‐DiHETE / 11,12‐EEQ	0.0157 ± 0.0297	0.0213 ± 0.0460	.761	
14,15‐DiHETE / 14,15‐EEQ	0.0438 ± 0.0611	0.0768 ± 0.1360	.761	
17,18‐DiHETE / 17,18‐EEQ	0.07161 ± 0.0836	0.1085 ± 0.1720	.855	
7,8‐DiHDPA / 7,8‐EDP	0.0912 ± 0.0956	0.0820 ± 0.1138	.391	
10,11‐DiHDPA / 10,11‐EDP	0.0924 ± 0.1188	0.0928 ± 0.1449	.903	
13,14‐DiHDPA / 13,14‐EDP	0.1745 ± 0.1935	0.1417 ± 0.1715	.502	
16,17‐DiHDPA / 16,17‐EDP	0.0329 ± 0.0354	0.0415 ± 0.0595	.583	
19,20‐DiHDPA / 19,20‐EDP	0.1952 ± 0.2757	0.2417 ± 0.3745	.542	

## 4 DISCUSSION

Our data demonstrate that all four subclasses of CYP epoxy metabolites and several LOX/CYP ω/(ω‐1)‐hydroxylase metabolites are increased by the hemodialysis treatment. We found that these changes are unlikely related to altered sEH activity. The data are also not related to alterations of plasma or red blood cell (RBC) fatty acid levels, in particular RBC n‐3 fatty acid status, which we demonstrated in our previous study (Gollasch, 2020). Despite significant changes in fatty acids signatures between healthy persons and CKD patients, we observed that hemodialysis does not alter plasma or RBC fatty acid levels to potentially explain the observed changes of oxylipins in the present study (Gollasch, 2020). Our data support the idea that 9,10‐EpOME, 12,13‐EpOME, 5,6‐DHET, and 5‐HETE are key markers to discriminate ESRD patients from healthy controls (Hu, 2018). It is unlikely that these changes occurred in response to chronic dialysis treatment since altered levels in 9,10‐EpOME, 12,13‐EpOME, 5,6‐DHET, and 5‐HETE levels were observed in patients with CKD before starting renal replacement therapy (Hu, 2018). While the ESRD patients in (Hu, 2018) were uremic Asians (eGFR 5–6 ml/min/1.73 m^2^) and recruited before beginning renal replacement therapy for fistula construction surgery, our patients were Caucasian ESRD patients undergoing regular, thrice weekly dialysis. Nonetheless, we observed similar changes in 9,10‐EpOME, 12,13‐EpOME, 5,6‐DHET, and 5‐HETE levels. However, our study revealed also other oxylipins that is, specific signature, which are up‐ or down‐regulated in plasma of the ESRD patients. The extent to which they exhibit beneficial or detrimental cardiovascular effects, possibly in metabolite‐interacting networks, remains to be explored. Furthermore, future studies can clarify whether specific underlying renal diseases may have specific oxylipin profiles to discriminate between ESRD patients.

### 4.1 CYP epoxy metabolites

Hemodialysis increased all four subclasses of CYP epoxy metabolites (9,10‐EpOME, 12,13‐EpOME, 9,10‐DiHOME, 12,13‐DiHOME, 5,6‐EET, 14,15‐EET, 8,9‐DHET, 11,12‐DHET, 8,9‐EEQ, 11,12‐EEQ, 14,15‐EEQ, 17,18‐EEQ, 14,15‐DiHETE, 17,18‐DiHETE, 7,8‐EDP, 10,11‐EDP, 13,14‐EDP, 16,17‐EDP, 19,20‐EDP, 7,8‐DiHDPA, 10,11‐DiHDPA, 13,14‐DiHDPA, 16,17‐DiHDPA, and 19,20‐DiHDPA). Although these changes are unlikely related to altered sEH activity, but rather to the dialysis treatment itself, reduced in vivo sEH activity in CKD/ESRD (Zhang, 2015) may have contributed to the increased accumulation of all four epoxy metabolite classes in our CKD patients, compared to the healthy control subjects.

### 4.2 EETs/DHETs

We demonstrated that hemodialysis increased EETs/DHETs levels, as detected for 5,6‐EET, 14,15‐EET, 8,9‐DHET, and 11,12‐DHET. Endothelial cells are reservoirs of EETs and the primary source of plasma EETs (Jiang, Anderson, & McGiff, 2010, 2012; Jiang, 2011; Schunck, 2017), which produce profibrinolysis and reduce inflammation, vascular tone, and blood pressure (Jiang, Anderson, & McGiff, 2010, 2012; Jiang, 2011). 5,6‐DHET as like 5,6‐EET can produce vasodilation (Hercule, 2009; Lu, 2001), which could contribute to the cardiovascular response during maximal exercise (Gollasch, 2019a). The mechanisms of how epoxides and diols are released from the tissues and eventually become constituents of circulating lipoproteins are largely unknown, making it difficult to explain our findings. Cells preferentially release DHETs while storing the EETs (Roman, 2002), suggesting that certain diols might be overrepresented in the circulating blood compared with the respective diol/epoxide ratios (Fischer, 2014). Our data support the idea that DHETs/EETs are attractive signaling molecules for cardiovascular effects in ESRD because they are potent vasodilators (Campbell & Fleming, 2010), which could counteract circulating vasoconstrictor substances during dialysis. Therapeutic sEH inhibition is considered a novel approach for enhancing the beneficial biological activity of EETs (Spector & Kim, 2015). However, presumably higher levels of EETs in blood and tissue in vivo may have also detrimental cardiovascular side effects (Gschwendtner, 2008; Hutchens, 2008; Wutzler, 2013). Of note, levels of all four EETs (5,6‐EET, 8,9‐EET, 11,12‐EET, and 14,15‐EET) were high in the ESRD patients compared to the control. The extent to which this increase has beneficial or detrimental cardiovascular effects remains to be explored.

### 4.3 EpOMEs/DiHOMEs

We observed increases in 9,10‐EpOME, 12,13‐EpOME, 9,10‐DiHOME, and 12,13‐DiHOME during dialysis. 9,10‐EpOME (leukotoxins A) and 12,13‐EpOME (leukotoxin B) were initially found to be generated by neutrophils during the oxidative burst to combat bacterial infection (Thompson & Hammock, 2007). Recent findings suggest that EpOMEs exhibit cardiodepressant (Fukushima, 1988; Siegfried, 1990; Sugiyama, 1987) and vasoactive properties, the latter by endothelial NO and O(2)(*‐) production (Okamura, 2002). Moreover, 9,10‐EpOME and 12,13‐EpOME can exhibit vasoconstrictor responses in severe cardiac ischemia (Dudda, Spiteller, & Kobelt, 1996; Siegfried, 1990) and could contribute to the cardiovascular response during maximal exercise (Gollasch, 2019a). New data suggest that DiHOMEs cause detrimental effects on postischemic cardiac function (Bannehr, 2019b; Chaudhary, 2013). Our data support the notion that increases in EpOMEs/DiHOMEs could affect cardiac ischemia and hemodynamics in dialysis patients.

### 4.4 EEQs/DiHETEs

We observed increases in 8,9‐EEQ, 11,12‐EEQ, 14,15‐EEQ, 17,18‐EEQ, 14,15‐DiHETE, and 17,18‐DiHETE during dialysis. While the putative biological functions of EEQs/DiHETEs have not received much attention, 17,18‐EEQ has been identified as a potent vasodilator, which seems to be even more potent than EETs (Hercule, 2007; Lauterbach, 2002). Their diols could contribute to the cardiovascular response during maximal exercise (Gollasch, 2019a). The mechanisms of how EEQs/DiHETEs are released from the tissues are largely unknown, making it difficult to explain our findings. Based on our calculations of diol/epoxide ratios, we have no evidence that the higher levels of 14,15‐DiHETE, 17,18‐DiHETE result from in vivo sEH enzyme activation. Nevertheless, the role of circulating EEQs/DiHETEs has yet to be integrated into a physiological and pathophysiological context. This is particularly important since drugs that mimic 17,18‐EEQ are viewed as novel promising drug candidates to overcome limitations of dietary EPA/DHA (C20:5 n–3/22:6 n–3) supplementation for cardiovascular health benefits (Schunck, 2017).

### 4.5 EDPs/DiHDPAs

We observed increases in 7,8‐EDP, 10,11‐EDP, 13,14‐EDP, 16,17‐EDP, 19,20‐EDP, 7,8‐DiHDPA, 10,11‐DiHDPA, 13,14‐DiHDPA, 16,17‐DiHDPA, and 19,20‐DiHDPA during dialysis. Little is known about the biological functions of EDPs/DiHDPAs. 16,17‐EDP and 19,20‐EDP are potent vasodilators in coronary, pulmonary, and mesenteric arteries. They lower blood pressure and exhibit cardioprotection by preservation of mitochondrial function (Morin, Fortin, & Rousseau, 2011; Schunck, 2017). Based on our calculations of diol/epoxide ratios, we have no evidence that the higher levels of DiHPAs metabolites observed in our study result from in vivo sEH enzyme activation. Our data indicate that both EDPs and DiHPAs metabolites are novel candidates for vasoactive substances potentially released by dialysis to affect hemodynamics in these conditions.

### 4.6 LOX/CYP ω/(ω‐1)‐hydroxylase metabolites

We found that hemodialysis increased several LOX/CYP ω/(ω‐1)‐hydroxylase metabolites (13‐HODE, 20‐HETE, 5‐HEPE, 15‐HEPE, 18‐HEPE, 19‐HEPE, 7‐HDHA, and 11‐HDHA). Little is known about the biological functions of those metabolites. 13‐HODE inhibits platelets (Buchanan, 1985) and could represent an important player in redox and immune homeostasis (Pecorelli, 2019; Vangaveti, 2018). 20‐HETE is a potent vasoconstrictor, which modulates intracellular signal transduction pathways in neovascularization (Chen, 2019) and in renal and cardiac ischemia‐reperfusion injury (Han, 2013; Hoff, 2011). Upregulation of 20‐HETE contributes to inflammation, oxidative stress, endothelial dysfunction, and increased peripheral vascular resistance (Waldman, 2016). 5‐HEPE promotes bovine neutrophil chemotaxis in vitro, but less potently than 5‐HETE (Heidel, 1989). 18‐HEPE is released by macrophages to inhibit cardiac fibrosis and inflammation in mice (Endo, 2014). Of note, 18‐HEPE appears to downregulate proinflammatory and pro‐proliferative factors, possibly via conversion to E‐series resolvins (Sapieha, 2011). These resolvins have effects similar to the D‐series resolvins, markedly reducing neutrophile infiltration, decreasing proinflammatory cytokines, and enhancing the resolution of inflammation (Sapieha, 2011). We have no evidence that hemodialysis affected resolvins, prostaglandins, thromboxanes, or other LOX/CYP ω/(ω‐1)‐hydroxylase metabolites in our patients (Shelmadine, 2017). Only few studies detected small changes in PGE2 or PGF2a (Losonczy, 1990; Schultze, 1984) and 5/12‐HETE levels (Dolegowska, 2012) 4‐HDHA is a mediator of antiangiogenic effects of n‐3 PUFAs (Sapieha, 2011). Since we found that the majority of LOX metabolites measured were not affected by dialysis, we suggest that these metabolites are unlikely to play important roles in this scenario.

## 5 CONCLUSIONS

To our knowledge, this is the first study to assess the impact of single hemodialysis treatment oxylipins in plasma using large‐scale lipidomics. We confirmed our hypothesis that the oxylipins status is influenced by hemodialysis treatment. Our data demonstrate that all four subclasses of CYP epoxy metabolites and a number of LOX/CYP ω/(ω‐1)‐hydroxylase metabolites are increased by the treatment. Moreover, ESRD patients undergoing regular dialysis show marked differences in plasma oxylipin profiles, that is, specific signatures, compared to control subjects. Future research is required to determine the contribution of the identified oxylipins in reducing the risk from CVD in patients with kidney disease.

## CONFLICT OF INTEREST

None.

## AUTHOR CONTRIBUTIONS

BG, MG, and FCL planned and designed the experimental studies. ID and MR performed the HPLC–MS spectrometry experiments. All authors contributed to the implementation and analyses of the experiments. BG drafted the article, and all authors, contributed to its completion.
